# Migration of superior vena cava stent

**DOI:** 10.1186/1749-8090-3-12

**Published:** 2008-03-10

**Authors:** Nitin B Bagul, Phillipa Moth, Narayan J Menon, Fiona Myint, George Hamilton

**Affiliations:** 1 Royal Free Hospital, London, UK

## Abstract

There has been a recent increase in the use of endovascular prostheses resulting in complex surgical and interventional complications not previously recognised. We report a case of Superior vena cava stenosis treated with a wallstent which migrated to the right atrium, necessitating a combined radiological and surgical approach to retrieve it.

## Introduction

There has been a recent increase in the use of endovascular prostheses resulting in complex surgical and interventional complications not previously recognised. We report a case of Superior Vena Cava Stenosis treated with a Wallstent which migrated to the right atrium, necessitating a combined radiological and surgical approach to retrieve it.

## Illustrative case history

A 78 year old haemodialysis patient was admitted under the care of renal team with a history of symptomatic bilateral upper limb and facial swelling unrelated to her renal impairment. In the past, her right groin had been irradiated for lymphoma and she had significant co-morbidity precluding any surgical intervention. She had duplex scanning, which showed evidence of Superior Vena Caval obstruction. She underwent angioplasty and Stenting of the Superior Vena Cava with a 10 mm × 46 mm Wallstent Uni endoprosthesis (Boston Scientific Corp., MA, USA). A right Internal Jugular Vascath was inserted proximal to the stent and good flows were achieved from all three lumen.

**Figure 1:**
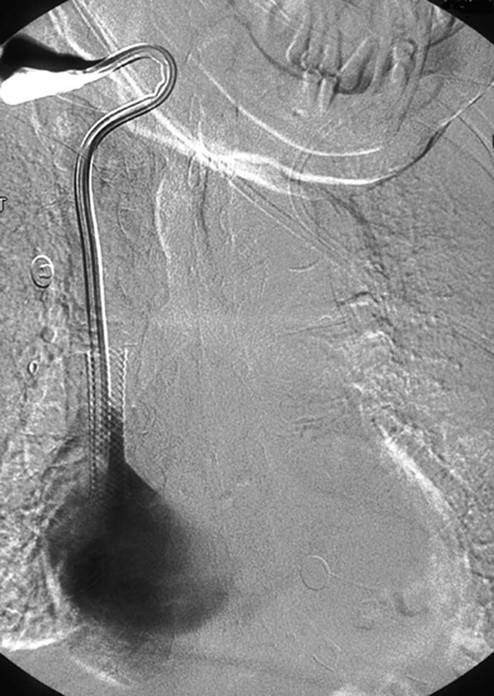
Right Internal Jugular Catheter and SVC stent in Situ.

**Figure 2:**
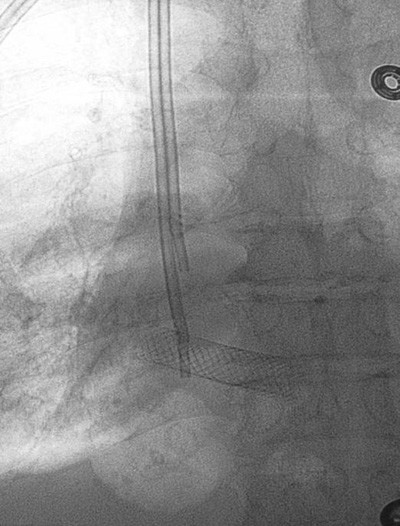
SVC stent lying transversely in right atrium.

Two weeks later since her supracaval venous access became blocked and a tunnelled Permacath was placed in the left femoral vein. An Amplatz superstiff wire was placed across the SVC stent through the pre-existing vascath. Under fluoroscopy, the line was removed and a peel-away sheath was introduced over the wire, to facilitate the introduction of a tunnelled Split-Cath. The intersect between the two different length lumina caught on the superior margin of the SVC stent instantly dislodging it. The stent moved into and lay horizontally in the right atrium [Fig 1, 2].

**Figure 3:**
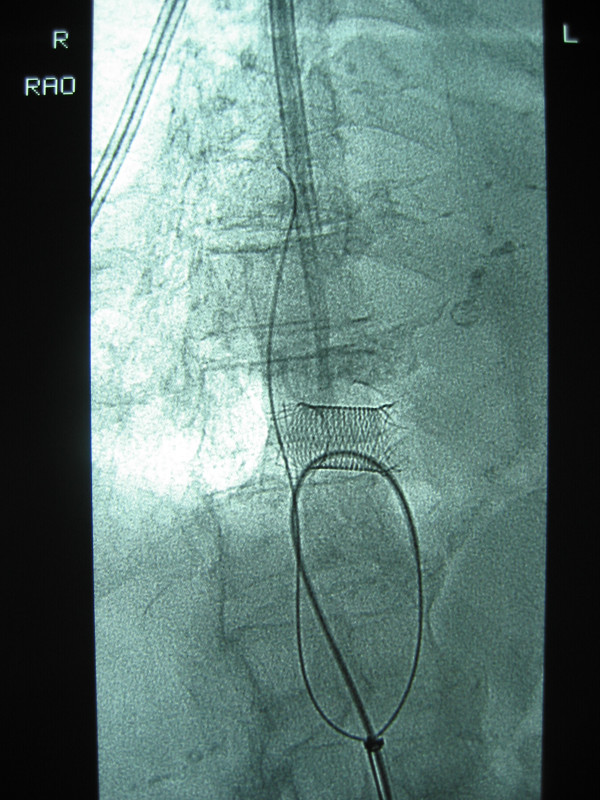
Stent caught in Terumo wire & Snare.

**Figure 4:**
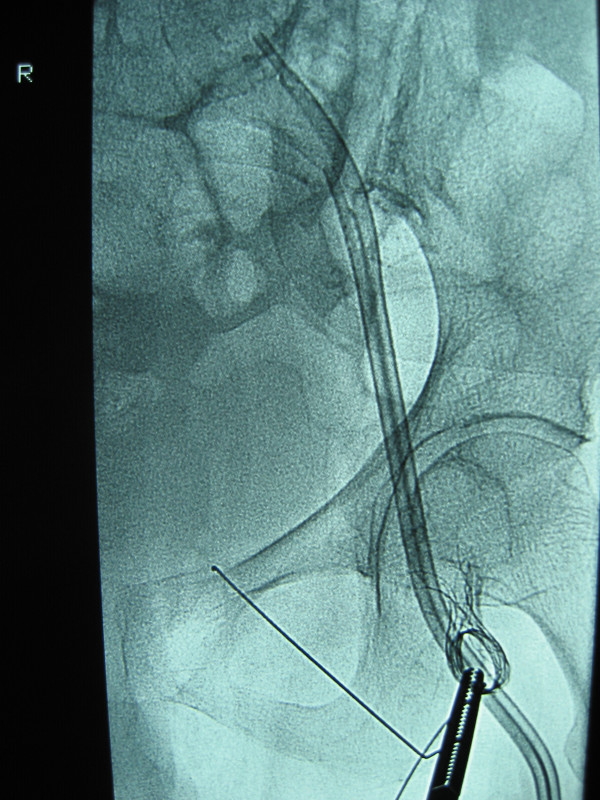
Stent pulled back in Common Femoral vein.

A 45 cm 10 Fr SuperArrow-flex percutaneous sheath was inserted in the left femoral vein, immediately superior to the tunnelled split-cath. Through this a 25 mm Amplatz gooseneck snare (ev3 Inc, MI, USA) was inserted and several futile attempts were made to snare the stent. A sidewinder catheter (Cordis Europa N.V) over an angled Terumo wire (Terumo Corp, Japan) was used to access the lumen of the stent. The Terumo wire, having passed through the length of the stent, was then snared through the gooseneck [Fig 3]. The snare was pulled into the sheath and a loop consisting of the sidewinder and Terumo wire was tightened, shortening the stent along its length. The constrained stent was pulled down into the left groin, the sheath and the sidewinder catheter were removed and the loop of the wire was held externally with a surgical artery clamp [Fig 4].

Since the left femoral access was precious and the radiological options were exhausted the vascular surgeons were consulted. The patient was too unfit to be anaesthetised and it was decided that the best option in her case was to try to retrieve the stent from the common femoral vein under local anaesthesia.

**Figure 5:**
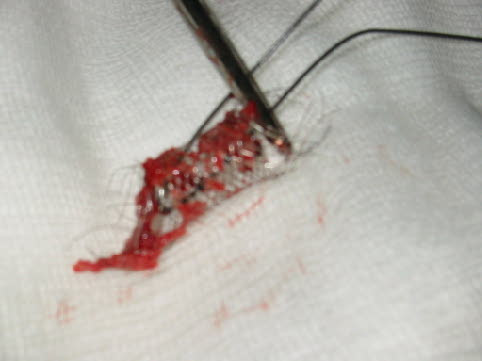
Stent retrieved surgically.

Under intravenous sedation and local infiltration, the common femoral vein was controlled proximal and distal to the venous access and through a venotomy the stent was retrieved [Fig 5]. The tunnelled line was still in-situ and functional. Subsequent venography showed no visible damage to the right atrium, inferior vena cava, iliac or femoral veins. On review months later, the patient continues to be dialysed through the femoral line with no known ill effect as a result of the reported complication.

## Discussion

With an increasing number of central venous access procedures in recent years in oncology, renal failure and Nutrition, the incidence of upper extremity venous occlusion is also more prevalent. It is estimated that as many as 40% of patients who undergo subclavian vein catheterisation eventually develop venous stenosis [1]. The postulated mechanisms involve turbulence, nonphysiologic increase in flow volume, platelet aggregation, fibrosis and stenosis of the lumen by central venous catheters [2].

Various interventional radiological procedures have evolved to treat central venous obstruction, such as balloon angioplasty, pharmacologic and mechanical thrombolysis, thrombectomy and stenting. The frequent use of stenting as therapy for venous occlusion is controversial. Dotter first described Stents in arterial system in 1969 [3]. Zollikofer in 1988 described the clinical applications in the venous system [4]. Stenting is thought to act as an adjuvant to venous angioplasty by limiting the elastic recoil in compliant veins, excluding the damaged and dissected vasculature and counteracting extrinsic compression. Both balloon-expandable stents like Palmaz stents and self-expandable flexible stents like Wallstents have been implanted in central venous stenoses [5], but because they mould better to the vessel wall, Wallstents are preferred by most Radiologists for restoring venous patency. A self-adjusting stent is advantageous since chronic venous occlusions may undergo progressive luminal enlargement after stent deployment [6].

Stenting symptomatic venous obstruction achieves temporary benefit but regular follow up and reinterventions may be required to maintain patency [7]. Primary venoplasty patency rates can be increased by 10% at 12 months by Stenting with a reduction in procedures for restenosis [8].

Migration or misplacement of endoprostheses is being recognised more often [9]. Stent migration can result in potentially serious complications such as lodgement in the right ventricle [10-12]. Percutaneous techniques for rescuing dislocated endovascular stents can be effective with few complications in most cases [13]. In extreme cases; displaced stents could be left in-situ with no disastrous consequences [14].

The increasing complexity of the complications arising from endovascular procedures often require the vascular interventionist to be innovative in order to rescue vascular access which is precious both for the patients and their physicians [15]. On this Occasion a combined radiological and Surgical Procedure was required.

Care must be taken where catheter are inserted through the lumina of such stents as dislodgement may result in due consequences.

## Competing interests

The author(s) declare that they have no competing interests.

## Authors' contributions

All authors were involved in the care of the patient. NBB and PM drafted the manuscript. NJM, FM, GH critically reviewed and improved the manuscript. All authors read and approved the final manuscript.

## Consent

Written informed consent was obtained from the patient for participation in this research.

A copy of the written consent is available for review by the Editor-in-Chief of this journal.
